# Assessment of the Effective Sensitivity of SARS-CoV-2 Sample Pooling Based on a Large-Scale Screening Experience: Retrospective Analysis

**DOI:** 10.2196/54503

**Published:** 2024-09-24

**Authors:** Jorge J Cabrera Alvargonzalez, Ana Larrañaga, Javier Martinez, Sonia Pérez Castro, Sonia Rey Cao, Carlos Daviña Nuñez, Víctor Del Campo Pérez, Carmen Duran Parrondo, Silvia Suarez Luque, Elena González Alonso, Alfredo José Silva Tojo, Jacobo Porteiro, Benito Regueiro

**Affiliations:** 1 Microbiology Department Complexo Hospitalario Universitario de Vigo Servicio Galego de Saude Vigo Spain; 2 Galicia Sur Health Research Institute (IIS Galicia Sur) Microbiology and Infectology Research Group Vigo Spain; 3 Centro de Investigación en Tecnologías, Energía y Procesos Industriales University of Vigo Lagoas-Marcosende Vigo Spain; 4 Applied Mathematics I Telecommunications Engineering School University of Vigo Vigo Spain; 5 Department of Preventive Medicine and Public Health Complexo Hospitalario Universitario de Vigo Vigo Spain; 6 Dirección Xeral de Saúde Pública Consellería de Sanidade Xunta de Galicia Santiago de Compostela Spain; 7 Dirección Xeral de Maiores y atención Sociosanitaria Conselleria de Politica Social e Xuventude Xunta de Galicia Santiago de Compostela Spain

**Keywords:** pooling, sensitivity, SARS-CoV-2, PCR, saliva, screening, surveillance, COVID-19, nonsymptomatic, transmission control

## Abstract

**Background:**

The development of new large-scale saliva pooling detection strategies can significantly enhance testing capacity and frequency for asymptomatic individuals, which is crucial for containing SARS-CoV-2.

**Objective:**

This study aims to implement and scale-up a SARS-CoV-2 screening method using pooled saliva samples to control the virus in critical areas and assess its effectiveness in detecting asymptomatic infections.

**Methods:**

Between August 2020 and February 2022, our laboratory received a total of 928,357 samples. Participants collected at least 1 mL of saliva using a self-sampling kit and registered their samples via a smartphone app. All samples were directly processed using AutoMate 2550 for preanalytical steps and then transferred to Microlab STAR, managed with the HAMILTON Pooling software for pooling. The standard pool preset size was 20 samples but was adjusted to 5 when the prevalence exceeded 2% in any group. Real-time polymerase chain reaction (RT-PCR) was conducted using the Allplex SARS-CoV-2 Assay until July 2021, followed by the Allplex SARS-CoV-2 FluA/FluB/RSV assay for the remainder of the study period.

**Results:**

Of the 928,357 samples received, 887,926 (95.64%) were fully processed into 56,126 pools. Of these pools, 4863 tested positive, detecting 5720 asymptomatic infections. This allowed for a comprehensive analysis of pooling’s impact on RT-PCR sensitivity and false-negative rate (FNR), including data on positive samples per pool (PPP). We defined *Ct*_ref_ as the minimum cycle threshold (*Ct*) of each data set from a sample or pool and compared these *Ct*_ref_ results from pooled samples with those of the individual tests (Δ*Ct^P^*). We then examined their deviation from the expected offset due to dilution [ΔΔ*Ct^P^* = Δ*Ct^P^* – log_2_]. In this work, the Δ*Ct^P^* and ΔΔ*Ct^P^* were 2.23 versus 3.33 and –0.89 versus 0.23, respectively, comparing global results with results for pools with 1 positive sample per pool. Therefore, depending on the number of genes used in the test and the size of the pool, we can evaluate the FNR and effective sensitivity (1 – FNR) of the test configuration. In our scenario, with a maximum of 20 samples per pool and 3 target genes, statistical observations indicated an effective sensitivity exceeding 99%. From an economic perspective, the focus is on pooling efficiency, measured by the effective number of persons that can be tested with 1 test, referred to as persons per test (PPT). In this study, the global PPT was 8.66, reflecting savings of over 20 million euros (US $22 million) based on our reagent prices.

**Conclusions:**

Our results demonstrate that, as expected, pooling reduces the sensitivity of RT-PCR. However, with the appropriate pool size and the use of multiple target genes, effective sensitivity can remain above 99%. Saliva pooling may be a valuable tool for screening and surveillance in asymptomatic individuals and can aid in controlling SARS-CoV-2 transmission. Further studies are needed to assess the effectiveness of these strategies for SARS-CoV-2 and their application to other microorganisms or biomarkers detected by PCR.

## Introduction

SARS-CoV-2, the virus responsible for the COVID-19 pandemic, has caused over 771 million infections and more than 6.9 million deaths worldwide [1].

To control the spread of the virus, it is essential to quickly detect as many infected individuals as possible and locate and test potential contacts [2-5]. Despite the initial debate [2], it was soon realized that testing only symptomatic individuals was insufficient, as nonsymptomatic individuals (including both asymptomatic and presymptomatic people) play a significant role in the transmission of SARS-CoV-2 [6,7]. Another important challenge was the occurrence of false-negative tests and their implications [8], which is especially significant in nonsymptomatic patients who may have low viral loads at the onset of infection. Although the likelihood of these false negatives is related to test sensitivity, it is always present. It was later described how the frequency of testing could be more important than its sensitivity [9], leading to the evaluation of concepts such as “the sensitivity of the test regimen” in the search for effective containment strategies [10]. All these findings indicate that for effective pandemic control, a “one-size-fits-all” approach was inadequate, and the key attributes of tests differ depending on whether they are used for diagnosis, detection, or surveillance [11]. Considering the results obtained after 2 rounds of screening all residents and workers at care homes in Galicia, Spain, via nasopharyngeal swabs (NPSs) and individual testing (involving more than 25,000 people from 306 Galician long-term care facilities), we evaluated a pooling strategy for the control of SARS-CoV-2 [12]. We decided to develop a new large-scale screening strategy that would increase the capacity and frequency of testing for nonsymptomatic individuals.

The combination of samples for SARS-CoV-2 detection has garnered attention for its ability to conserve testing resources and increase the number of samples that can be processed, despite the increased detection limit associated with pool size [13-18]. Thus, validating the pooling assay and instrumentation is critical to reducing false-negative results [19-21]. In any case, using pooling as a screening technique rather than as a diagnostic method minimizes its shortcomings [9]. The Centers for Disease Control and Prevention (CDC) Interim Guidance [22] establishes the differences between diagnostic, screening, and surveillance testing and the specific requirements for each pooling application. The US Food and Drug Administration (FDA) June 2020 COVID-19 update [23] outlined Emergency Use Authorization (EUA) steps for the broad screening of asymptomatic individuals and testing using a sample pooling technique.

To minimize the impact of the reagent shortage through the application of pooling techniques, a second limitation in increasing the number of people tested was sample collection. Since the beginning of the pandemic, NPSs have been the reference sample for the diagnosis of SARS-CoV-2, despite requiring qualified personnel and strict protocols to avoid contagion. The results are highly dependent on sample collection, which is considered highly invasive and unpleasant for patients [24]. Therefore, for large-scale screening, an alternative sample that allows for simple self-sampling (ensuring adherence) without loss of quality was necessary. Research on the detection of other respiratory viruses in saliva [25,26] and previous work by our group with saliva and SARS-CoV-2 [27] piqued our interest in this approach. Although there was no validated technique for using saliva samples at the beginning of this work and serious doubts about its suitability for diagnosing SARS-CoV-2, various studies had postulated saliva as the preferred alternative sample [24,28-31]. Erratic results were observed when comparing saliva and NPSs [24,32-34]. This variability could be related to significant differences in what different studies defined as “saliva,” including variations in the type of saliva collected, its preservation [32,35], the presence of enzymes [36], and viscosity [37]. All these factors significantly impact the sensitivity of the technique. Nevertheless, the advantages of using saliva for large-scale diagnosis, despite its possible limitations, seem to justify its use [38-40]. In May 2020, the FDA approved the first test for SARS-CoV-2 detection in saliva [41], and in May 2021, a technical report from the European Centre for Disease Prevention and Control (ECDC) concluded that saliva can be used for symptomatic patients and for repeated screening of asymptomatic individuals [42]. As with saliva, although pooled samples were widely used during the pandemic, there was no FDA- or CE (Conformité Européenne)-approved option for their use at the time this work began. It was not until April 20, 2021, that the FDA issued an amendment [43] to authorize additional indications for EUA-approved kits for use on pooled samples, but under very strict conditions. These restrictive conditions have resulted in only 4 tests obtaining this approval, none of which are included in this study.

This paper aims to evaluate the performance of pooling in large-scale screening for SARS-CoV-2 detection in asymptomatic individuals, particularly in critical areas such as care institutions, hospitals, universities, and key industries. Building on our previous work, where we proposed the implementation of pooling strategies in these settings [12], we hypothesize that increasing testing frequency in these groups, facilitated by pooling techniques on saliva samples, can enable early detection of asymptomatic individuals.

## Methods

### Study Design

This work is a retrospective analysis of real-time polymerase chain reaction (RT-PCR) results for SARS-CoV-2 from 888,665 saliva samples using Dorfman pooling. From August 2020 to February 2022, 56,515 pools of varying sizes and 54,194 individual samples were tested, with 39,928 samples excluded for various reasons. The samples included health care workers, industrial workers, and employees from other organizations involved in periodic screening, as well as participants in “massive screenings” or “pharmacy screenings” who underwent isolated tests. Massive screenings were organized by the regional public health department to control virus spread in specific areas of the region. In these screenings, self-collection saliva kits were sent to specific municipalities for distribution to the general population. Additionally, pharmacies throughout the region were supplied with kits, allowing individuals to voluntarily participate. Exclusion criteria were being symptomatic or having close contact with COVID-19; in such cases, individuals were referred to the conventional diagnostic pathway (individual PCR and NPS sample). To ensure participants were fully informed about the potential limitations of the method, these details were clearly outlined in the agreement signed by each participating entity and in the participant information sheet provided (Multimedia Appendix 1). To ensure participants were fully informed of the method’s potential limitations, these details were explicitly outlined in the agreements signed by each entity and in the information sheets provided (Multimedia Appendix 1).

### Collection and Registration Samples

Participants were instructed to collect their sample immediately after waking and before engaging in any activities that could reduce the presence of the virus (eg, eating, drinking, brushing teeth, chewing gum, smoking), and only if they were asymptomatic and had no known close contacts. If any participant did not meet these criteria, they were referred for diagnosis using NPSs and individual tests, which we referred to as the conventional method. Participants collected at least 1 mL of saliva in a Vircell Transport Medium-2 1-mL (12 × 80 mm) tube saliva collection device immediately after waking, without eating, drinking, smoking, or chewing gum (dilution factor ≤1/2). The kit allowed for self-sampling and included a unique barcode label for sample identification. We began the project using the GeneFix Saliva DNA/RNA Collector-GFX, the only manufacturer with a suitable device and sufficient capacity to meet our needs. Although initial tests were satisfactory, we soon encountered issues with inadequate long-term RNA stability at room temperature in certain samples. Although this was manageable in the initial phases of the study, which involved local samples, it proved unsuitable for scaling up the project to include samples from the entire region of Galicia. For this reason, we decided to eliminate virus neutralization and use a new device developed in collaboration with Vircell (Transport Medium-2 1-mL—12 × 80 mm tube). This device is designed to collect saliva in a viral transport medium, ensuring long-term virus stability and the preservation of its RNA [32]. It also allows for the recovery of the virus if necessary for subsequent studies.

After collection, participants had to scan their personal QR code and then the sample barcode with their smartphone camera and confirm the registration [44,45]. This process ensured that samples were processed in a pseudonymized manner, with the only information associated with each sample being the QR code and its reporting site.

### Preanalytical Steps

All samples arrived at the laboratory in green Beckman Coulter 14-mm 50-position racks, which were directly loaded into AutoMate 2550 (Beckman Coulter). The AutoMate 2550 device performed tasks such as sample reception (ie, verifying that the sample was expected by the system), optical measurement of sample volume, uncapping, and sorting the samples into 24-position HAMILTON tube carriers for subsequent processing or into the error area. Samples were directed to the error area if they had incorrect registration, erroneous barcode readings, or if a low volume was detected. All error samples were reviewed by a microbiologist and, if possible, corrected or rejected with the corresponding error noted. Samples with a confirmed total volume of less than 1.6 mL were rejected due to potential loss of sensitivity from sample dilution. All actions associated with each sample performed by the AutoMate 2550 device were logged and sent to the Laboratory Information System Modulab (Werfen, S.A.).

### Pooling

The racks with individual samples that passed the preanalytical stage were manually transferred to the Microlab STAR (HAMILTON) and managed using the HAMILTON Pooling software with the “Pooling—without archive plates” method and a parallel pipetting strategy to minimize time consumption. The Microlab STAR platform is divided into 54 equal tracks, with the deck layout for our method consisting of 12 tracks for 2 CO-RE tip carriers and 42 tracks for 24-position tube carriers, including 40 for samples and 2 for pools. The Microlab STAR we used had 8 pipetting channels with fully configurable movements. We chose to use joint movements, with all 8 heads pipetting samples in the first step and then into pool tubes in the next step. This approach optimized processing times and maximized sample throughput, although it meant that consecutive samples were assigned to different pools.

Total Aspiration and Dispense Monitoring (TADM) tolerance bands were carefully set to exclude samples with high viscosity, ensuring a homogeneous mixing of all saliva within the pool. If the pipetting system detected viscosity exceeding the threshold limit set in TADM, the sample was excluded from its respective pool. Subsequently, these samples were treated with a mucolytic agent by adding 2 drops of BD BBL MycoPrep reagent and allowing it to act for at least 30 minutes before being reprocessed in a new STAR run. Samples with adequate viscosity levels were incorporated into pools, while those that remained above the viscosity limit were rejected. It is important to clarify that the use of mucolytic agents, such as BD BBL MycoPrep Reagent, is not included in the approved working protocols for the RT-PCR assays used in this study. We chose to use this agent following internal validation, during which we did not observe increases in *Ct* values in positive samples after treatment under the conditions of our study.

To assess the equipment time requirements for different tasks and strategically adjust the laboratory work schedule when the required time approaches or exceeds the laboratory’s operating hours, we used an Excel (Microsoft Corp.) file (Multimedia Appendix 2) where we could input the sample load and its expected prevalence. The final laboratory setup included 2 AutoMate (Beckman Coulter), 3 Microlab STAR (HAMILTON), 3 Microlab STARlet (HAMILTON), and 3 CFX96 (Bio-Rad Laboratories) instruments, enabling theoretical processing capacities ranging from 8000 to 15,000 samples per day for prevalences between 0.5% and 5%. The standard pool size was 20 samples (100 µL per sample), but the pool size was reduced to 5 (250 µL per sample) when prevalence exceeded 2% in any group. We also utilized the HAMILTON option “Incomplete pools are tolerated” since December 2020, which allows a pool tube to hold fewer samples than the preset size if there are not enough available samples to complete the last pool or if a pipetting error occurs. All actions associated with each sample performed on the Microlab STAR were logged and, since December 2020, sent to the Laboratory Information System Modulab (Werfen, S.A.).

**Figure 1:**
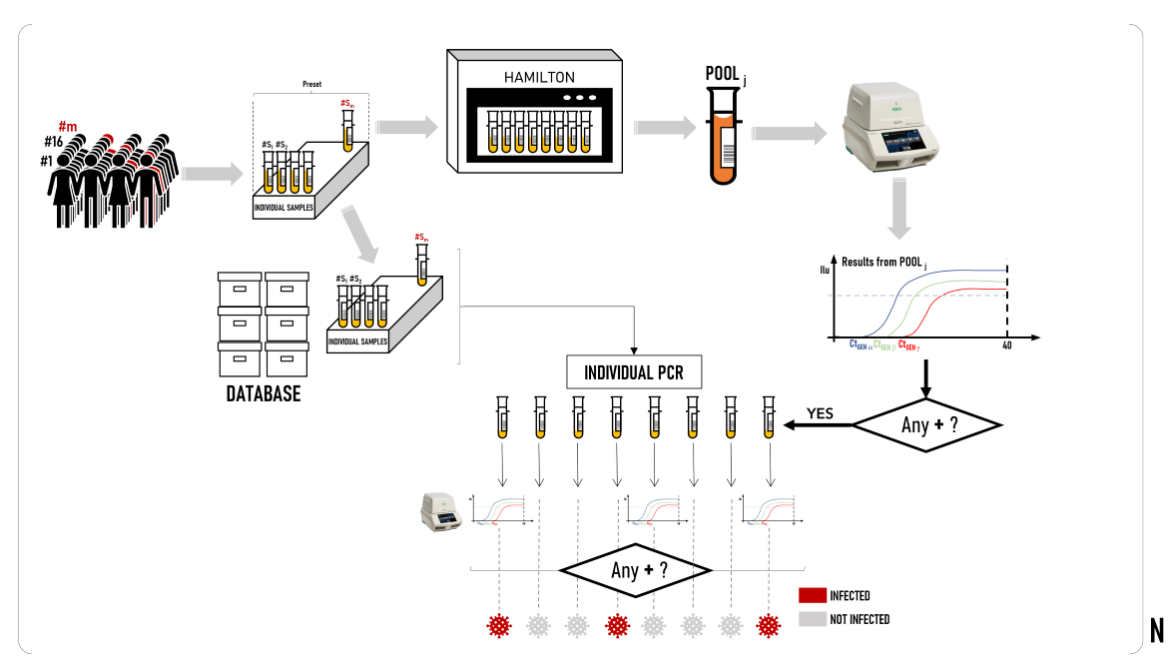
Schematic diagram of the pooling process. Starting with a population sampling of *m* individuals (where the red individuals symbolize an infected person), a total of *N* pools were formed, with each composed of the selected preset number. Afterward, RT-PCR was performed, and a result was obtained in which the luminescence of each target gene was analyzed and it was determined whether the sample was positive (ie, infected). If it was positive, the pool was undone, and an individual PCR was performed on the members of the pool who were detected to be positive. RT-PCR: real-time polymerase chain reaction.

When the Microlab STAR run was finished, the pool carriers were sent to the Microlab STARlet for the next step, while the sample carriers were loaded into the AutoMate 2550 device for archiving in red Beckman Coulter 14-mm 50-position racks (Figure 1).

### Nucleic Acid Extraction and SARS-CoV-2 Detection

All individual samples and pools were processed in the same way. Nucleic acid extraction was performed on a Microlab STARlet IVD platform (HAMILTON) using the STARMag 96×4 Universal Cartridge Kit (Seegene Inc.). To detect SARS-CoV-2, we used the Allplex SARS-CoV-2 Assay (Seegene Inc.), a multiplex RT-PCR assay designed to detect the RdRp, S, and N genes specific to SARS-CoV-2; the E gene for all Sarbecoviruses, including SARS-CoV-2; and an internal control. The manufacturer declares a limit of detection of 50 copies per reaction for this assay. From August 2021 to February 2022, we switched to the Allplex SARS-CoV-2 FluA/FluB/RSV assay. This assay includes targets for the detection of influenza A virus (FluA), influenza B virus (FluB), and respiratory syncytial virus (RSV); 3 targets for SARS-CoV-2 (N gene, RdRp gene, and S gene); and endogenous and exogenous internal controls. The manufacturer declares a limit of detection of 50 copies per reaction for SARS-CoV-2 and 100 copies per reaction for FluA, FluB, and RSV. This RT-PCR step was conducted on a CFX96 system (Bio-Rad Laboratories), with analysis performed using the Seegene Viewer software (Seegene Inc.). According to the manufacturer’s instructions, *Ct* values lower than 40 were considered detected, while values equal to or greater than 40 or marked as not applicable were considered undetected. Any pool with 1 or more detectable targets was considered positive, and the individual samples within such pools were analyzed in the same manner as the pools.

### Data Analysis for Individual and Pooled PCR Results

In this study, each pool of *n* samples that shows positive results is resolved into *n* individual tests. For each sample that tests positive, we have 2 sets of *Ct* values: 1 for the pool and 1 for the individual test. Although *Ct* values should not be regarded as quantitative measures of the virus amount in the sample or even in the patient, in our case, because both tests are performed in the same laboratory with the same equipment and the same test procedure (apart from differences related to sample preparation), we can use both sets of *Ct* values to analyze the impact of pooling on the test results.

To perform the analysis, we compiled a database that included the *Ct* value for each target gene for both pool and individual samples, pool size (*N*), and the number of positive samples per pool (PPP). We will refer to *Ct*_ref_ as the lowest *Ct* value among the target genes that tested positive. As all analytical steps are identical, *Ct*_ref_ will be useful for assessing sensitivity and precision among different targets, as well as the impact of pooling on the test’s performance.

We define here Δ*Ct^I^_G_*_(_*_i_*_)_ as the difference between the number of cycles that took the *i*th gene [*G*(*i*)] in the individual PCR to reach its threshold value and the minimum of all *Ct* values used in the PCR (*Ct*_ref_), where the subscript *i* stands for “individual.” Hence:

Δ*Ct^I^_G_*_(_*_i_*_)_ = *Ct^I^_G_*_(_*_i_*_)_ – *Ct*_ref_ = *Ct^I^_G_*_(_*_i_*_)_ – min[*Ct^I^_G_*_(_*_i_*_)_]; *i*=1, …, 4

Consequently, Δ*Ct^I^_G_*_(_*_i_*_)_ must be positive for all genes except one, which will be 0. Similarly, we define Δ*Ct^P^_G_*_(_*_i_*_)_ as follows, where *P* stands for “pool”:

Δ*Ct^P^_G_*_(_*_i_*_)_ = *Ct^P^_G_*_(_*_i_*_)_ – *Ct*_ref_ = *Ct^P^_G_*_(_*_i_*_)_ – min[*Ct^I^_G_*_(_*_i_*_)_]; *i*=1, …, 4

In this case, the difference is calculated between the pooled result and the minimal number of cycles in the PCR test, with *Ct*_ref_ serving as an indicator of the viral load in the individual sample. The value of Δ*Ct^P^_G_*_(_*_i_*_)_ can be decomposed into 2 terms: one representing the expected displacement due to the dilution in the pool (where *N* stands for the pool size) [46], and the other representing the difference in cycles of the specific target gene, ΔΔ*Ct^P^_G_*_(_*_i_*_)_.

Δ*Ct^P^_G_*_(_*_i_*_)_ = log_2_(*N*) + ΔΔ*Ct^P^_G_*_(_*_i_*_)_

**Figure 2:**
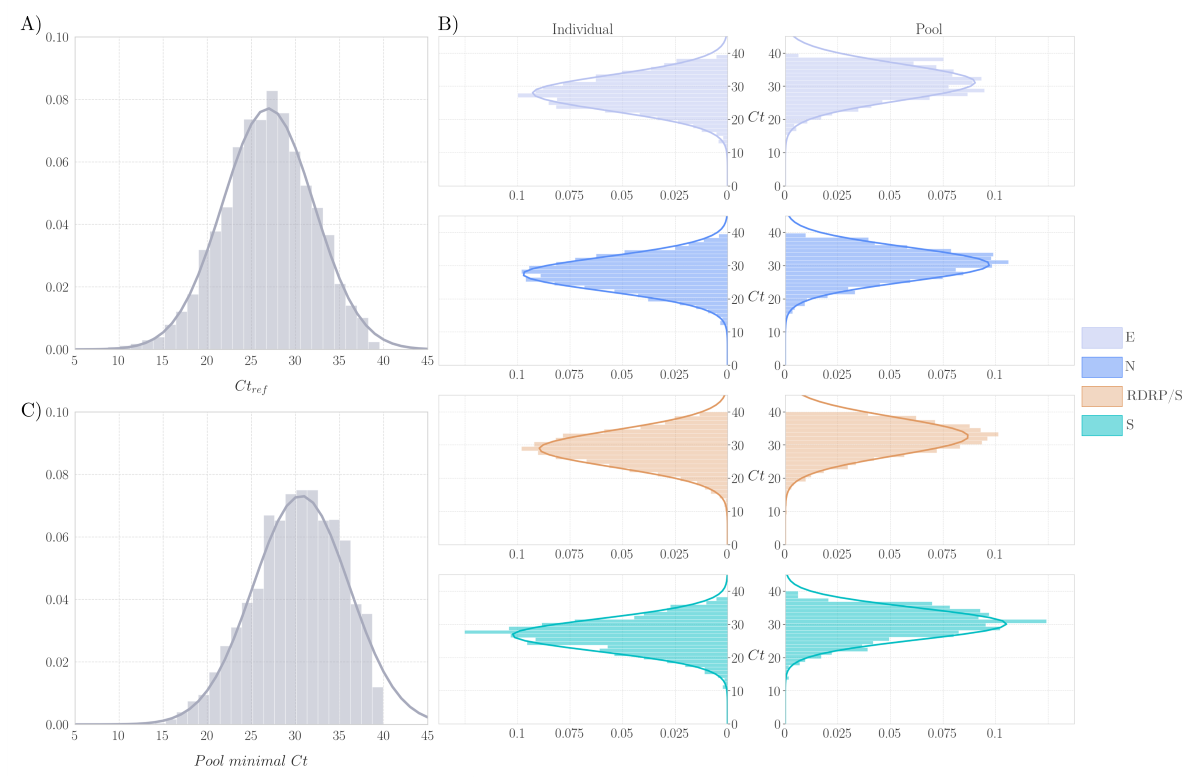
Pooling versus individual results (Galicia, Spain, data collected between August 2020 and February 2022; N=887,926 samples; retrospective analysis of SARS-CoV-2 infection). This compound figure includes the distributions of minimal *Ct* values for (A) individual tests and (C) sample pooling, and (B) breakdown of the *Ct* distributions for each of the genes (E, N, RdRp, and S) on the individual test (left) and within the pool (right). In such decomposition, individually, there are genes that tend to deviate to higher *Ct* values (such as RdRp), with gene S generally marking the positive detections.

**Figure 3:**
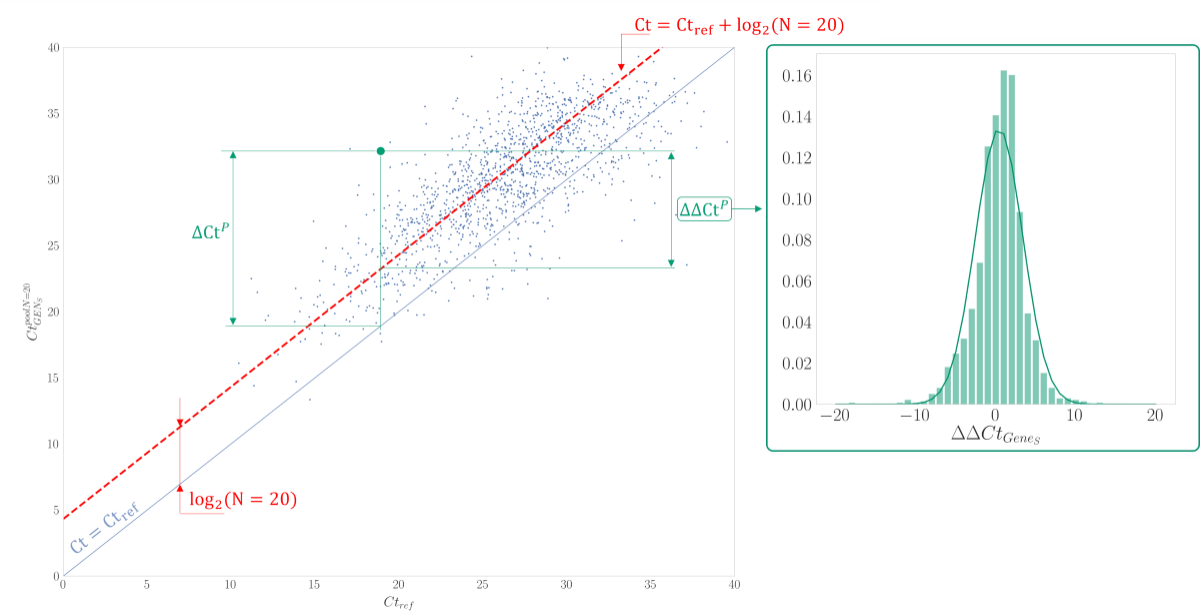
ΔΔ*Ct^P^_G_*_(_*_i_*_)_ concept. (Galicia, Spain, data collected between August 2020 and February 2022; N=887,926 samples; retrospective analysis of SARS-CoV-2 infection). Explanation of the concept of ΔΔ*Ct^P^_G_*_(_*_i_*_)_ and its relationship with ΔΔ*Ct^P^_G_*_(_*_i_*_)_ for gene S and pool size 20 (as an example). This differentiation is crucial for assessing the true deviation in infection detection due to the intrinsic deviation of log_2_(*N*) inherent to the chosen pool size (*N*). This is evident through the distribution of ΔΔ*Ct^P^_G_*_(_*_i_*_)_, which demonstrates a dispersion centered around 0 and illustrates instances where certain cases fall ahead of or behind the reference threshold cycle (*Ct*) value.

Finally, with 928,357 individual samples tested in 56,126 pools, which identified approximately 5720 positive samples, we can statistically infer the sensitivity loss due to pooling. To do so, the following hypotheses were proposed:

1. The population screened by pooling follows the normal distribution depicted in Figure 2A.
2. Pooling affects the test through dilution and scatter. As shown in Figure 3, dilution results in an overall displacement in the result equal to log_2_(*N*), while each gene’s result scatters according to a Gaussian distribution [ΔΔ*Ct^P^_G(i)_*].
3. The displacement experienced by each gene tested in the pool is independent of the displacement experienced by the other genes.
4. According to our standard procedure, a pool is interpreted as positive when at least one gene reaches the threshold before the completion of 40 cycles.
5. In a worst-case scenario, we will assume that no 2 or more individuals share the same pool.

Based on these hypotheses, the probability of a false-negative result [*P*(FN)] for the *i*th gene in a pool of *M* individuals can be calculated as the probability that the *i*th gene in the pool requires more than 40 cycles to reach the threshold, given that the pool contains a single positive individual with a viral load characterized by *Ct*_ref_:

*P*(FN) = *P*[*Ct*^P^_G(_*_i_*_)_(*Ct*_ref_) > 40] = *P*[ΔΔ*Ct^P^_G_*_(_*_i_*_)_ > 40 – *Ct*_ref_ – log_2_(*N*)]

For example, the probability of failing to detect gene S in an individual sample with a *Ct*_ref_ of 30 within a pool of 16 [log_2_(16) = 4] is the probability that gene S will experience a ΔΔ*Ct* greater than 6.

Assuming the normal distribution of the samples (hypothesis 1) and that only 1 gene needs to test positive (hypothesis 4), the overall probability of a false negative for a specific *Ct*_ref_ can be defined as follows:




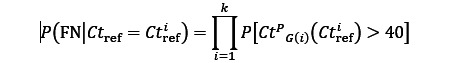




This means that for a pooled test with 1 sample having *Ct*_ref_=*Ct*_ref_*^i^* to result in a false negative, all target genes (*i*=1, …, *k*) in the test must simultaneously fail, where *k* represents the total number of genes.

Finally, the overall false-negative rate (FNR) of the pool methodology will be the combination of the FN probability for each *Ct*_ref_ and the observed population distribution:




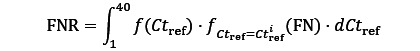




Hence, depending on the number of genes used in the test and the size of the pool, we can evaluate the FNR and effective sensitivity (1 − FNR) of the test configuration.

### Outcome Communication

Once validated, the sample results were sent to middleware for pseudonym reversal. Negative results were labeled as “not detected” to differentiate them from those obtained via conventional diagnosis, which are reported as “negative.” Additionally, each result included a disclaimer stating “test negativity does not exclude SARS-CoV-2 infection” for further clarification. These results were then communicated to individuals via SMS text messages (if opted), while positive results were reported to the Dirección General de Salud Pública (General Directorate of Public Health) and to the reporting site health responsible. They would inform the patient, and a new sample would be taken from them and all their high-risk contacts using the conventional method (NPS and individual test), aligning pooling and conventional diagnostic testing.

### Ethics Approval

The study received approval from the Galician Network of Research Ethics Committees (protocol number 2021/022) and adhered to the principles of the Declaration of Helsinki. All methodologies were implemented following the appropriate guidelines and regulations, with patient data pseudonymized. The data set used, along with the waiver of informed consent, was approved by the Galician Network of Research Ethics Committees. Explicit authorization was granted by the same network to use the pseudonymized data set and conduct the analysis for this publication without reverting the pseudonymization, ensuring compliance with ethical standards.

## Results

### Global Insights

**Figure 4:**
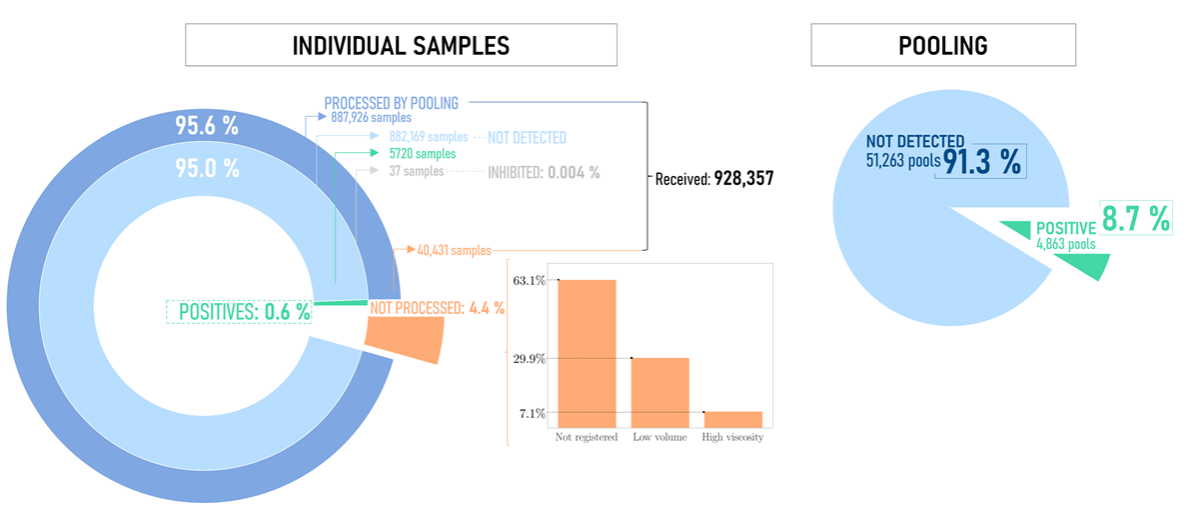
Global insights. The overall results of the analysis of individual and pool tests were evaluated. The results have been compartmentalized based on the samples that have undergone processing through pooling and those that have been discarded due to noncompliance with established criteria. Of the samples that were fully processed, a differentiation was made between those that were nondetected and those that were positive (ie, infected). Additionally, a representation of all the pools that were executed is provided, and they have been categorized into those that were nondetected and those that were positive and further individually analyzed.

Between August 2020 and February 2022, our laboratory received a total of 928,357 samples from 345,826 different QRs (individual for each person). Of these, 95.64% (887,926/928,357) were fully processed, while the remaining 4.36% (40,431/928,357) encountered issues that prevented their full processing. Additionally, 15,473 samples were registered but not received. The remaining samples were pooled in Microlab STAR into 56,126 pools of varying sizes, with 4863 of these pools testing positive. Subsequently, each of the 53,196 samples from the positive pools was processed individually, resulting in 5720 positive results (Figure 4).

We experienced punctual peaks of over 12,000 samples per day and maintained constant working volumes exceeding 4000 samples per day. The mean response time for all fully processed samples was approximately 16 (SD 12) hours.

### Pooling Insights

In this study, an overall positivity rate of 8.66% (4863/56,126) was observed in the pools. Of the 4863 positive pools, 97.20% (4727/4863) contained at least one positive individual sample, while 2.80% (136/4863) did not show any positive results in individual sample tests. These 136 false-positive pools generally had high *Ct* values and positivity for only 1 or 2 targets. Upon retesting, both the individual samples and the original pools were found to be negative. This observation highlights that the incidence of false-positive pools is significantly influenced by our permissive positivity threshold (any target with a *Ct*≤40). This threshold, established by the manufacturer, was maintained to maximize sensitivity for pooled testing. However, because a false-positive pool only leads to individual tests on samples that ultimately turn out to be negative, the overall impact on laboratory-reported results is negligible, with only a minor effect on the number of tests conducted and associated costs.

**Figure 5:**
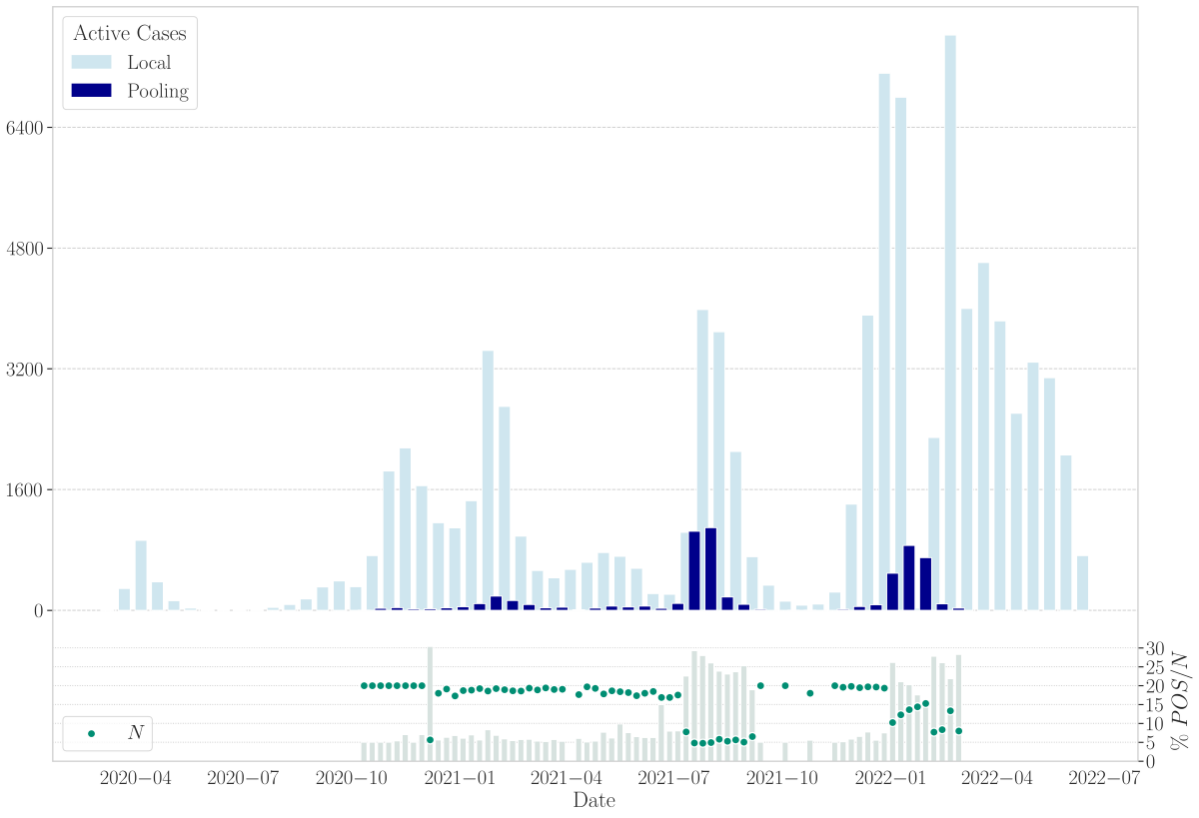
Positivity evolution (Galicia, Spain, data collected between August 2020 and February 2022; N=887,926 samples; retrospective analysis of SARS-CoV-2 infection). Comparative analysis of the distribution of infected cases during the pandemic in the local area of Vigo (Spain; light blue) with the positives detected in our laboratory (dark blue) in temporal increments of 2 weeks. Furthermore, a histogram is included below, which illustrates the progression of pool sizes (ie, *N*) in relation to the percentage of positives for each pool size (ie, %POS/*N*) throughout the pandemic.

Depending on the pandemic phase and the source of the samples (eg, positive clusters), some pools included multiple positive samples. Specifically, 14.60% (835/5720) of positive individual samples were part of pools with at least two positive samples (PPP≥2). Figure 5 illustrates that the daily number of positive cases detected followed the pandemic waves’ sequence. A similar trend was observed with PPP, reflecting the impact of varying pandemic waves on sample positivity.

**Figure 6:**
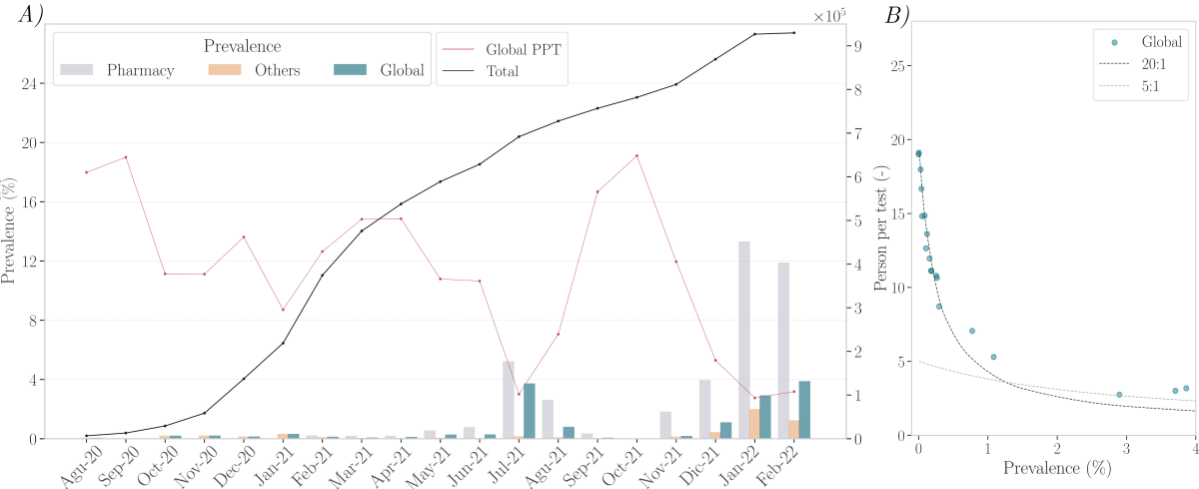
Evolution of the COVID-19 pandemic throughout the study period (Galicia, Spain, data collected between August 2020 and February 2022; N=887,926 samples; retrospective analysis of SARS-CoV-2 infection). (A) The number of cases analyzed is shown (solid black line); additionally, the number of PPT (solid red line) and the prevalence sampled in pharmacies (gray bar), other sources (orange bar), and globally (green bar) are shown. (B) The relationship between the PPT and the global prevalence is shown with the theoretical performance under the 20:1 and 5:1 protocols given as a reference. PPT: persons per test.

As illustrated in Figure 5, during several periods of the pandemic, the pool size was reduced from the preset 20 to 5 due to high positivity rates. The histogram confirms that these reductions corresponded with a higher percentage of positive pools when the pool size was 5. Furthermore, as shown in Figure 6, during the fifth and sixth waves, prevalence increases were not uniform across groups. Notably, samples received from pharmacies exhibited higher prevalence values compared with other sources. Consequently, the preset pool size of 5 was utilized specifically for samples from periods with higher positivity rates. Despite this, the preset size of 20 remained the most frequently used, and a notably higher number of positive cases were detected in pools of size 5. Additionally, due to the pipetting system’s configuration, individual samples could result in pools of sizes smaller than the intended size.

### Reference Cycle Threshold (Ctref)

**Figure 7:**
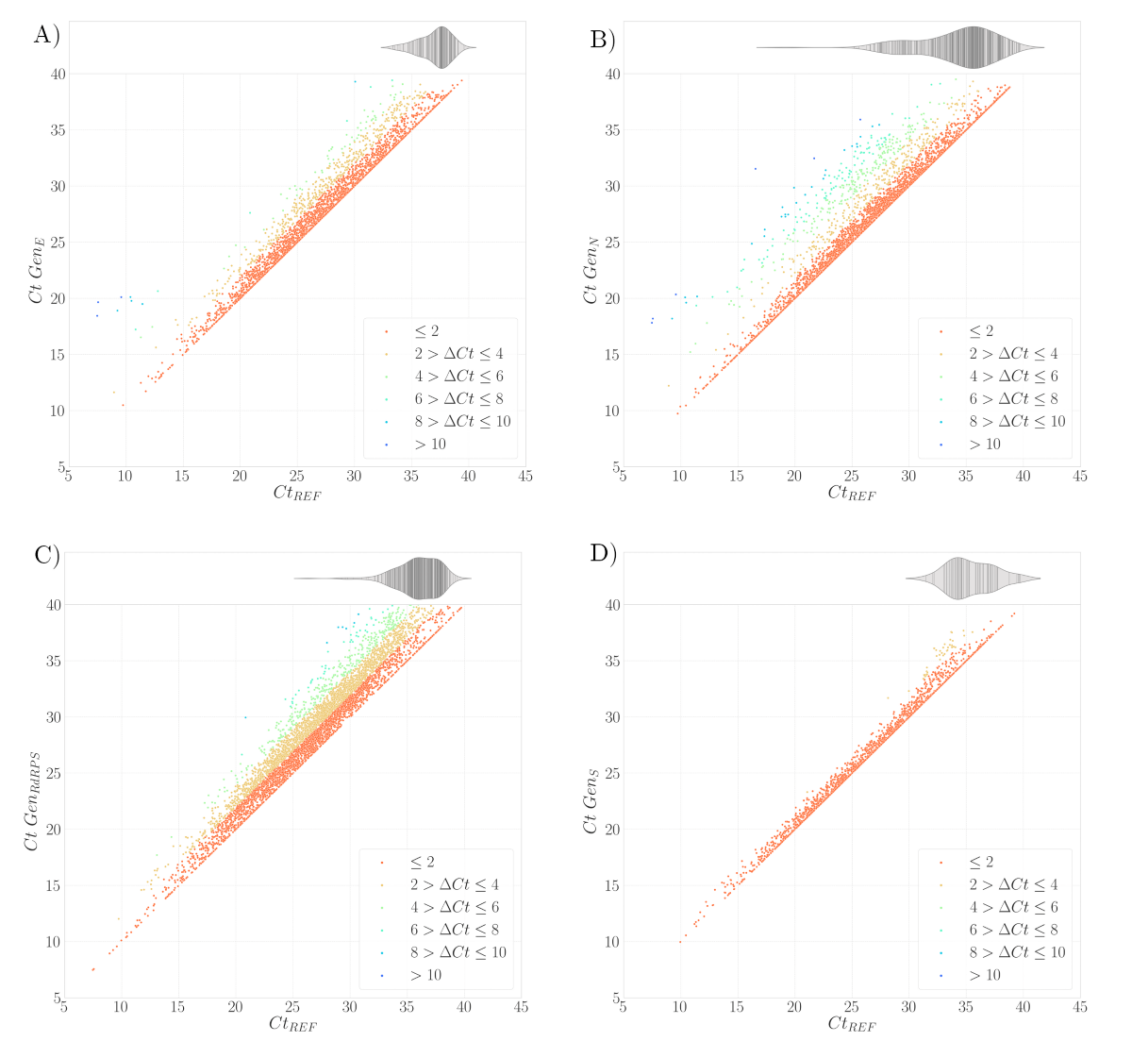
Individual *Ct* versus *Ct*_ref_ (Galicia, Spain) between August 2020 and February 2022 (N=887,926 samples; retrospective analysis of SARS-CoV-2 infection). A scatter plot showing the *Ct* value of each gene on the individual test versus the minimal *Ct* (ie, *Ct*_ref_), colored by the vertical distance between such values, which is known as Δ*Ct*. The violin plots shown at the top of each subfigure are those cases in which the individual gene (either E, N, RdRPS, or S) failed to detect a positive sample. Gene S shows a lower dispersion in the results in comparison with gene N, which not only has a higher dispersion of *Ct* but also has a higher distribution of failures reaching low values of *Ct*_ref_.

As shown in Figure 7, the scatter plot illustrates the *Ct* values of each gene compared with the minimum *Ct* value, denoted as *Ct*_ref_. By definition, no *Ct* value can be lower than *Ct*_ref_ for the sample. The plot reveals that most results are close to *Ct*_ref_, with gene S (Figure 7D) displaying exceptionally low dispersion. The vertical distance from the line representing *Ct*_ref_ to each point indicates the number of additional cycles required for that gene in the individual sample to reach the detection threshold. As 40 is the maximum limit, some genes in the sample may not even reach the threshold value before the test is stopped. These genes are considered to have “failed” to detect the virus in the sample and are represented by the horizontal violin graph on top of each graph. As shown, such “failures” typically occur when the sample *Ct*_ref_ is high. Notably, gene N shows some “failures” even when the virus presence in the sample is high, indicated by a low *Ct*_ref_.

### Pooled Versus Individual PCR

As previously stated, the main concern with sample pooling is the reduction in sensitivity due to sample dilution. Initially, a thorough analysis of the results will be conducted to assess, from a macroscopic perspective, whether any significant influences can be identified.

Figure 2A shows the distribution of *Ct*_ref_ values across individual samples, which follows a normal distribution (μ=26.88, σ=5.14, *P*=.007 [α=.01]). As the PCR procedure was stopped at 40 cycles, the data are truncated at this value. The same pattern is observed at the gene level (Figure 2B) and for the minimal *Ct* of the pool (Figure 2C), with both distributions fitting a normal curve (µ=30.65, σ=5.45, *P*=.12, α=.01). When samples are pooled, *Ct* distributions shift toward higher values, a shift that is evident at the gene level as shown by the lateral subplots.

**Figure 8:**
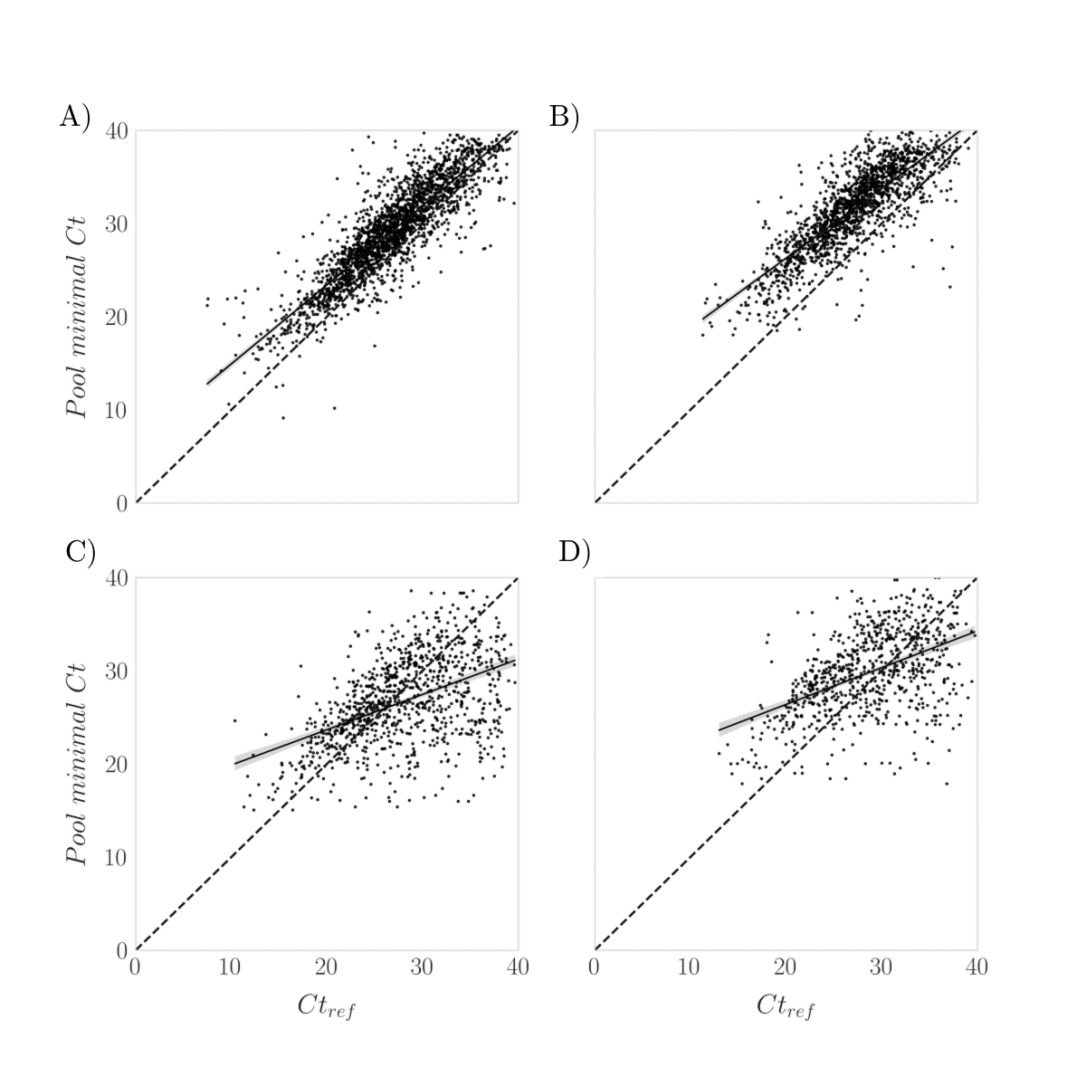
Pooling minimal *Ct* versus *Ct*_ref_ (Galicia, Spain, data collected between August 2020 and February 2022; N=887,926 samples; retrospective analysis of SARS-CoV-2 infection). Comparison of individual *Ct*_ref_ (x-axis) and pool minimal *Ct* (y-axis) for all pools with (A) PPP=1 and small pool size (N<6), (B) PPP=1 and large pool size (N>15), (C) PPP>1 and small pool size (N<6), and (D) PPP>1 and large pool size (N>15). The correlation between the 2 minimum Ct values is especially apparent for those pools that contained a single positive (top) and even more so for those of smaller size (A). However, as the viral load increases in the sample pool, this correlation vanishes. PPP: positive samples per pool.

Figure 8 illustrates the correlation between the minimal *Ct* of the pool and the *Ct*_ref_ of individual positive samples. Four distinct groups are classified based on pool size (small or large) and the number of positive samples within the pool, that is, PPP (PPP=1 and PPP>1). As shown in Figure 8A and 8C, when the pool is small (*N*<6), the increase in the number of cycles, indicated by the distance between the cloud of points and the x=y line, is smaller compared with larger pools (*N*>15). Nonetheless, there is a strong correlation between *Ct*_ref_ and the minimal *Ct* in the pool. However, when PPP exceeds 1, this correlation is no longer valid (see Figure 8C and 8D). In such instances, while dilution from nonpositive samples in the pool leads to a shift toward higher *Ct* values in the PCR test results, the sample with the highest viral load can still cause the threshold to be reached earlier compared with the number of cycles required by a sample with a lower viral load in its individual test. As a result, many pools may have a minimal *Ct* lower than the *Ct*_ref_ of some individual samples.

In our study, the observed Δ*Ct^P^* global was 2.24, compared with 3.31 for pools with 1 positive sample per pool (PPP1), with ΔΔ*Ct^P^* values of –0.88 and 0.23, respectively (Multimedia Appendix 3).

Figure 3 illustrates the concept of ΔΔ*Ct^P^_G_*_(_*_i_*_)_ for all samples with a pool size of 20 and the target gene S. As depicted, when the cycle displacement is corrected, the pooled results scatter around 0, displaying a bell-shaped distribution.

### Pooling Overall Effective Sensitivity

**Figure 9:**
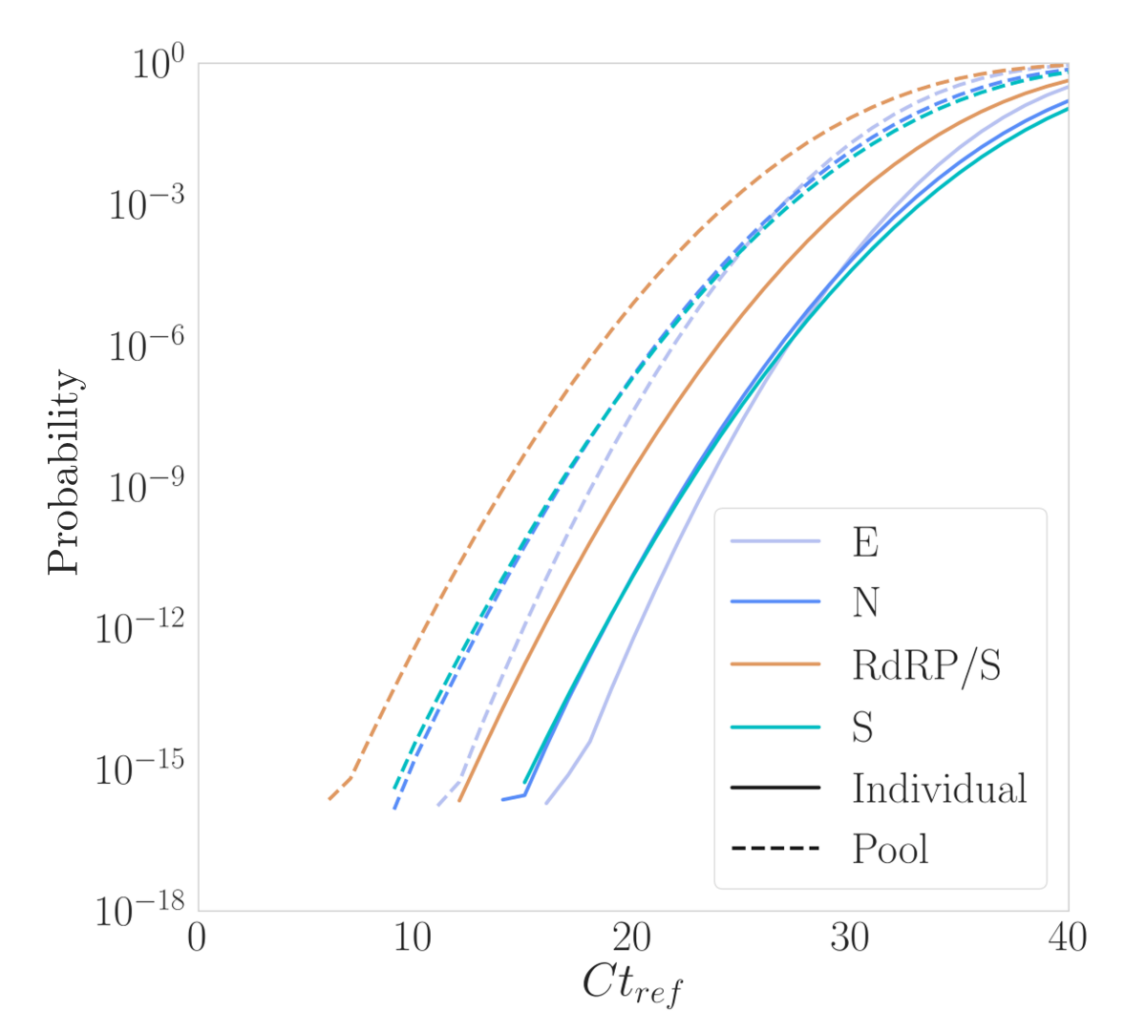
False negative (FN) versus *Ct*_ref_. The probability of an FN for each test is presented, with the results for individual tests (solid line) and a reference pool size of 20 (dashed line), separated by gene (E, N, RdRp/S, and S). As anticipated, the probability of an FN is higher for pooled samples compared with individual tests and higher when it gets closer to 40, as expected.

Based on the data compiled during our COVID-19 pandemic experience, Figure 9 illustrates the probability of a false negative for each gene used in the test. As shown, when the viral load in an individual sample is high (*Ct*_ref_=20), the probability of a false negative for any gene is in the range of [ppm] to [ppb]. At lower viral loads (*Ct*_ref_=30), while the probability of a false negative for each gene in an individual test is around .001, the probability of a false negative for a pool of 20 (as shown in Figure 9) increases to approximately .1.

As shown in Table 1, a pool of 20 will have an effective sensitivity of 90% [*P*(FN)=.1] for individual samples with *Ct*_ref_ in the range of 30-32, and an effective sensitivity of 99% for individual samples with *Ct*_ref_ in the range of 26-29.

Table 2 provides sensitivity values for pool sizes of 5, 20, and 32 with 3 genes (E, N, and RdRp/S) and 4 genes (E, N, RdRp/S, and S) simultaneously used. As shown, the sensitivity remains above 99% even for a pool size of 32 when all 4 target genes are used.

Applied to our case, where the maximum pool size was set to 20 with 3 target genes, we statistically observed that the effective sensitivity remained above 99%.

**Table 1:** *Ct_ref_*^a^ values for different false-negative probability levels comparing the results for an individual test and a sample pool size of 20.

*P*(FN)^b^	Individual test					Pool (*N*=20)			
	E	N	RdRp/S	S	E		N	RdRp/S	S
0.1	37.53	39.00	36.11	39.80	32.28		33.25	30.64	33.87
0.01	34.33	35.24	32.36	35.97	29.12		29.50	26.99	30.03
.001	32.07	32.50	29.66	33.11	26.78		26.83	24.22	27.16

^a^*Ct*_ref_: minimum cycle threshold.

^b^*P*(FN): probability of false negative.

**Table 2:** Effect of the number of targets and pool size on the effective sensitivity. Pooling performance for different pool sizes is compared with the performance for the individual test.

Test and target genes						False-negative rate (%)		Effective sensitivity (%)
**Individual**								
	E	N/A^a^	N/A	N/A	0.078		99.922	
	E	N	N/A	N/A	0.012		99.988	
	E	N	RdRp/S	N/A	0.0053		99.995	
	E	N	RdRp/S	S	5.7 × 10^–04^		99.999	
**Pool (*N*=5)**								
	E	N/A	N/A	N/A	1.80		98.20	
	E	N	N/A	N/A	0.31		99.69	
	E	N	RdRp/S	N/A	0.17		99.83	
	E	N	RdRp/S	S	0.04		99.96	
**Pool (*N* =20)**								
	E	N/A	N/A	N/A	4.83		95.17	
	E	N	N/A	N/A	1.34		98.66	
	E	N	RdRp/S	N/A	0.91		99.09	
	E	N	RdRp/S	S	0.37		99.63	
**Pool (*N*=32)**								
	E	N/A	N/A	N/A	6.44		93.56	
	E	N	N/A	N/A	2.03		97.97	
	E	N	RdRp/S	N/A	1.44		98.56	
	E	N	RdRp/S	S	0.66		99.34	

^a^N/A: not applicable.

### Pooling Efficiency and Economic Implications

From an economic perspective, the primary concern is pooling efficiency, typically assessed by the number of individuals who can be tested with a single test, referred to as persons per test (PPT). The PPT can be determined as shown in Table 3.

**Table 3:** Pooling efficiency.

Efficiency	Total count
Total samples, n	928,357
Total pools/PCRs^a^ for pools, n	56,126
Positive pools, n	4863
PCRs for individual samples of positive pools, n	53,196
Positive samples, n	5720
Total PCR, n	109,322
Prevalence of fully processed samples, %	0.64
Prevalence pools, %	8.7
Persons per test of fully processed samples	8.122
Pooling cost (in euros^b^)	3,279,66
Individual processing cost (in euros)	26,637,78
Pooling saving cost (in euros)	23,358,12

^a^PCR: polymerase chain reaction.

^b^€1=US $1.09.

As expected and shown in Figure 6A, the number of tests saved due to pooling is higher during periods of low prevalence. At a prevalence near 0 with a default pool size of 20, the average PPT approaches 20, as almost all pools test negative and no additional testing is needed. However, even a slight increase in prevalence has a considerable impact on the average PPT. This effect can be mitigated to some extent by adjusting the pool size used. The increase in prevalence was notably higher in pharmacies due to the nature of punctual screening. Consequently, we adjusted the pool size from 20 to 5 for these samples. During peak periods, the laboratory received over 100,000 samples, with a peak of nearly 153,000 samples in February 2021. As shown in Figure 6B, the impact of prevalence on PPT was significant; even a slight increase to 0.5% in prevalence caused the PPT to drop from nearly 20 to less than 10. As shown in Figure 6B, when prevalence slightly exceeds 1%, the standard 20:1 protocol becomes less effective compared with the 5:1 ratio, as it requires more individual testing of pools.

## Discussion

### Principal Findings

Our 19-month study, which involved over 928,000 saliva samples, demonstrates the practical scalability and efficiency of saliva pooling as a screening strategy for SARS-CoV-2, particularly among asymptomatic populations in critical areas such as care institutions, hospitals, and universities. The main findings indicate that while pooling can reduce the sensitivity of RT-PCR, effective sensitivity can remain high, above 99%, with appropriate pool sizes and multiple target genes. We observed a considerable economic benefit, with savings exceeding 20 million euros (US $22 million). To address the operational complexity of sample pooling, we implemented automated systems from the outset. Initially, a quality system ensured proper pipetting and homogenization on a small scale. As we scaled up, we developed a comprehensive system for complete traceability. This contrasts with manual pooling and nonspecialized equipment, which, while acceptable in some contexts, lacks the same precision and error reduction. Establishing this laboratory required adapting existing commercial systems (AutoMate and Microlab STAR) to meet our specific needs. We also collaborated in developing a new registration platform, a saliva sampling device, and IT solutions at Modulab (Werfen, S.A.) to ensure full traceability. To our knowledge, this was the first system with these characteristics available at the time we started the project.

The system allows for the detection of 3 types of incidents in the samples received: samples without registration (25,492/928,357, 2.75%), low volume (12,089/928,357, 1.30%), and high viscosity (2850/928,357, 0.31%). Although the incidence of errors can be considered high, it can be justified by the self-sampling and self-registration processes, as well as the lack of supervision during these phases. In any case, a drop in the percentage of these errors was observed over time in the groups that performed the cyclical screening. We believe this is due to the practice and familiarity acquired by the participants. Special mention should be made of the evaluation of viscosity in Microlab STAR (HAMILTON) using the TADM technology. We adjusted the TADM settings to identify samples that had excessive viscosity, thereby preventing them from becoming part of a pool. A low dispersion in the viscosity values of the samples that form a pool ensures a homogeneous and stable mixture, reducing the risk of false negatives during the RT-PCR phase. This occurs because pipetting an aliquot of the pool in which not all samples are equally represented can lead to inaccuracies. Additionally, high-viscosity saliva samples have been associated with a decrease in the sensitivity of SARS-CoV-2 RT-PCR [37]. Therefore, eliminating these samples can help preserve the sensitivity of the pooling process.

The logistics of our project involve users collecting their own samples and delivering them to their work centers. All samples from multiple work centers (540 organizations) must then be transported to the pooling laboratory. Consequently, RNA stability in saliva samples is a major concern for the success of the project. Initially, we evaluated commercially available saliva collection media (GeneFix Saliva DNA/RNA Collector-GFX) and found that RNA stability was inadequate in certain samples. Testing positive NPS samples with and without saliva revealed that rapid RNA degradation occurred in the presence of specific saliva, possibly due to varying concentrations of endonucleases [35,36]. To address this problem, we initially considered inactivating these enzymes using RNA stabilizers, heat, or microwave methods [46,47], but these options were deemed too complex. Instead, we chose to forgo virus neutralization and implemented a new device developed in collaboration with Vircell. This device collects saliva in a viral transport medium, ensuring the long-term stability of the virus and natural protection of its RNA, as previously described [32].

Focusing on positive samples, we observed a low overall prevalence in this study (*P*=.006). This finding is expected, given that a high percentage of the samples came from groups undergoing cyclical screening of asymptomatic individuals. As illustrated in Figure 6, prevalence data were quite similar across different groups during the second wave (October 2020 to January 2021). From the third to fourth wave, the prevalence in the “others group” was substantially lower than that in the samples received from pharmacies. This difference is likely due to the more frequent biweekly or weekly screening in the “others group” compared with the sporadic testing conducted at pharmacies. As previously described [9-11], screening frequency is a key factor in determining the efficacy of SARS-CoV-2 control, even when pooling testing is used [47]. During the fifth and sixth waves, the prevalence of samples collected from pharmacies increased compared with other sources. This increase was likely due to a loss of control over participant inclusion criteria, which were supposed to be asymptomatic individuals without evidence of close contact [48]. The surge in positivity was primarily linked to pharmacy samples, which was driven by the overwhelming demand during these waves and the saturation of standard microbiological diagnostic systems. As a result, we reduced the preset pool size from 20 to 5 for samples from pharmacies, which improved efficiency compared with processing all samples with the larger pool size (Figure 6B).

An important aspect of this study is the data set of 4863 positive pools of varying sizes, paired with 5720 positive individual results. This data set allows for an in-depth analysis of the impact of pooling on RT-PCR results, particularly regarding effective sensitivity and FNR. Our analysis (see Table 2) revealed an effective sensitivity of 99.83% for pools with 5 samples and 99.09% for pools with 20 samples. Correspondingly, the FNRs were 0.0170 and 0.0916 for pools of 5 and 20 samples, respectively. It is important to highlight that these results were calculated for PPP1 pools, representing a “worst-case” scenario. In pools with more than 1 positive sample, the probability of detecting a positive result increases, as previously described [49,50] and illustrated in Figure 2. This effect is evident when comparing the global Δ*Ct^P^* global (2.24) with PPP1 (3.31), and the corresponding ΔΔ*Ct^P^* values of –0.88 and 0.23, respectively (Multimedia Appendix 3). Therefore, the maximum FNR associated with our pooling system would not exceed 1%, maintaining an adequate sensitivity (≥99%) for screening purposes [22]. This sensitivity is further enhanced when screening is performed frequently on a population with low pretest probability (asymptomatic individuals without known risk contacts) [8].

The inclusion of multiple targets for SARS-CoV-2 is intended to mitigate sensitivity losses or false negatives caused by potential mutations affecting primer binding sequences [51,52]. However, this approach is likely to reduce false negatives even in the absence of such mutations. Multiplex PCR is a complex diagnostic system that can be susceptible to various errors, potentially leading to variations in *Ct* values or the failure to detect 1 or more targets. Given the lack of previous studies on this issue, we considered it necessary to investigate the impact of the number of targets on RT-PCR sensitivity and FNRs in both individual and pooled testing (Table 2). The results indicated a significant decrease in FNRs with each additional target, regardless of whether individual or pooled samples were processed and irrespective of pool size. When focusing on the performance of each target individually (Figures 5 and 7 and Table 1), the S gene stands out in our data set. However, this performance might differ if another data set were used. It is important to note that the S gene showed significantly higher *Ct* values compared with other targets with the alpha variant [53,54]. However, with the assay we used (Allplex SARS-CoV-2/FluA/FluB/RSV assay), an increase was observed for the N gene [55]. These effects are not reflected in our data because we transitioned to the S gene in August 2021, a time when the alpha variant was no longer prevalent in our region [56]. Less obvious variations in *Ct* values for different SARS-CoV-2 targets have also been described using machine learning algorithms, potentially related to distinct characteristics of major SARS-CoV-2 variants [57]. For these reasons, we can infer that using RT-PCR techniques with multiple targets for the same virus can enhance diagnostic sensitivity. This approach not only mitigates the risk of false negatives due to mutations affecting primer binding sites but also reduces the likelihood of all targets being negative simultaneously. Therefore, combining pooling techniques with diagnostic tests that have multiple targets can minimize sensitivity loss due to dilution, enhancing the reliability of these tests [58].

Finally, the pooling system demonstrated high efficiency in the study population with a PPT greater than 8. This resulted in substantial savings on reagents and associated costs, totaling over 23 million euros (US $25 million). This project aimed to develop a comprehensive diagnostic system that integrates RT-PCR techniques with pooling strategies, enabling frequent large-scale screening in critical areas. This approach helps limit SARS-CoV-2 transmission within these groups, conserves testing resources, and ensures an adequate response time. Although economic savings were not the primary motivation for this project, reducing associated costs has been crucial for its long-term sustainability.

Vaccination against SARS-CoV-2 has been successful in many countries, with over 13 million doses administered [59]. This has contributed to reducing morbidity and mortality among the infected. However, it has not eliminated transmission and has increased the proportion of asymptomatic individuals [60-63]. Asymptomatic individuals remain a critical population in transmission control [64]. Immunocompromised patients with persistent infections may play a significant role in the emergence of mutations characteristic of variants of concern [65,66]. Even if vaccinated, these patients might present increased opportunities for resistance to antibodies or T-cell escape [63]. This issue could be particularly significant in high-transmission environments, such as care homes [67,68]. Additionally, the uneven distribution of vaccines worldwide hampers the control of transmission [1]. Despite the extensive knowledge gained about SARS-CoV-2, our understanding remains limited, and making long-term predictions without considering a broad range of possibilities would be imprudent [69]. Therefore, systems such as the one we present, which enable rapid and effective access to large populations, can serve as valuable tools for widespread surveillance and responding to new challenges posed by SARS-CoV-2 [63].

Current engineering solutions for sample processing in clinical diagnostic laboratories face limitations. Most robotic and automated systems are designed with linear geometry, which restricts their ability to create dynamic circuits for the intelligent and individualized handling of specific samples. These limitations may arise not only from the capabilities of the automation systems themselves but also from the constraints in developing or modifying software to meet specific needs associated with new tasks. Additionally, there is a lack of integration between automated systems from different providers, which hinders potential synergies and the creation of continuous flow systems. This results in the need to sequence processes across disconnected stages, often requiring unnecessary human intervention and increasing the risk of nontraceable errors. This constraint limits the potential for “smart” pooling [70] and alternative protocols for processing positive pools [71]. It underscores the need for a new architecture in automated systems for clinical laboratories, one that can accommodate these new strategies and adapt to future innovations.

### Limitations

The main limitations of the study are our inability to conclusively establish the effectiveness of large-scale saliva pooling as a screening strategy for controlling the transmission of SARS-CoV-2 due to the inherent complexities involved in such an evaluation. It is important to note that the positive sensitivity data reported are specific to the population studied and are conditioned by the inclusion of only those samples that tested positive in the pool. Nevertheless, we believe that the approach proposed for calculating effective sensitivity holds promise, though further studies are needed to fully validate these findings. Additional limitations are the challenges in comparing saliva with NPS data and assessing the system’s ability to detect influenza A/B and RSV. The data suggest that in locations where frequent cyclical screening was implemented, the first positives were detected earlier in the outbreak. The limited mortality rate recorded in care homes seems to support this idea. In cases where *Ct*_ref_<35, positives were detected in the first NPS. Additionally, following the reagent change from August 2021 to February 2022, we identified 315 positives for RSV, 92 for influenza A, and 0 for influenza B in saliva pooling. These findings coincided with the circulation of these viruses in our region during that period [72].

### Conclusions

Our study demonstrates that, despite the inherent reduction in RT-PCR sensitivity due to sample pooling, highly effective sensitivity can be maintained using multiple target genes and appropriate pool sizes. This approach can be tailored to the epidemiological situation and adjusted according to the specific population being tested. New studies are needed to evaluate the effectiveness of these strategies in screening or surveillance for SARS-CoV-2 [73], as well as their application to other microorganisms, such as respiratory viruses in vulnerable populations, hepatitis C [74], sexually transmitted infections [75,76], *Mycobacterium tuberculosis* [77], the next “Disease X” [78-80], and other PCR-diagnosed biomarkers. The operational and economic advantages of our pooling system highlight its significant potential, which could be further enhanced by improving the architecture of automated systems in clinical laboratories.

The implications of these findings are substantial, potentially enabling a more proactive approach to public health management by optimizing early detection, outbreak control, and reducing health care and economic burdens. Extending these methodologies to other microorganisms and biomarkers could significantly impact epidemiological surveillance and diagnostics, facilitating faster and more effective responses to both current and future public health challenges.

In conclusion, integrating these strategies into existing health care systems would enhance pandemic response capabilities and offer a robust platform for addressing other health issues. These developments underscore the need for an interdisciplinary and collaborative approach in research and clinical practice to maximize their benefits.

## Appendix

Multimedia Appendix 1 Documents for participants.

Multimedia Appendix 2 Lab schedule assessment tool.

Multimedia Appendix 3 Comparison between Δ*Ct^P^* and ΔΔ*Ct^P^* Global vs PPP1.

## Abbreviations

CDC: Centers for Disease Control and Prevention
CE: Conformité Européenne
*Ct*: cycle threshold
*Ct*_ref_: minimum cycle threshold
ECDC: European Centre for Disease Prevention and Control
EUA: Emergency Use Authorization
FDA: US Food and Drug Administration
FNR: false-negative rate
NPS: nasopharyngeal swab
P(FN): probability of false negative
PPP: positive samples per pool
PPP1: pools with 1 positive sample per pool
PPT: persons per test
RT-PCR: real-time polymerase chain reaction
TADM: Total Aspiration and Dispense Monitoring

## Data Availability

All data associated with this study are available in the published paper (including its multimedia appendices) and in the complete raw data collected from the laboratory information system, accessible online [81].
