# Supplementary material for: Assessment of the Effective Sensitivity of SARS-CoV-2 Sample Pooling Based on a Large-Scale Screening Experience: Retrospective Analysis
Source: JMIR Public Health Surveill. 2024 Sep 24;10:e54503. doi: 10.2196/54503 (PMC11462102; doi:10.2196/54503)
Supplement: Multimedia Appendix 1 [file publichealth_v10i1e54503_app1.docx]

**Solicitud de cribado preventivo de personas asintomáticas mediante técnicas de procesamiento agrupado “Pooling”**

La detección de Coronavirus SARS-CoV-2 mediante RT-PCR utilizando técnicas de agrupamiento de muestras “pooling” para su procesamiento, puede ser útil como **estrategia preventiva** aplicada en poblaciones sanas. Su aplicación en grandes grupos de personas puede permitir la **detección de individuos pre y asintomáticos**, de forma rápida y económica.

Su principales limitaciones son:

1. Disminución en la sensibilidad analítica asociada a este tipo de procesamiento, esta disminución **puede producir un aumento en la tasa de falsos negativos** en comparación con el técnicas de procesamiento individual.
2. Se trata de una estrategia preventiva, **NO es una técnica diagnóstica**, y su utilidad se encuentra en proceso de validación. Por lo que cualquier persona que presente síntomas compatibles con COVID-19 debe ser excluida del cribado.

Los individuos que no presentan síntomas no son objeto de estudio mediante los protocolos actuales, salvo que se relacione como posible contacto de un caso diagnosticado. Es por ello que este tipo de cribado, a pesar de sus limitaciones, puede aportar beneficios en términos de prevención a la población incluida, respecto a otras en las que no se realice este tipo de estrategia.

Yo, …………………………..……………………….. en calidad de …………………..…………...de la Empresa/Entidad …………………………………. solicito la realización del cribado preventivo de SARS-Cov-2 mediante técnicas de *pooling* a los trabajadores que voluntariamente quieran entrar a formar parte del mismo. Asimismo, declaro conocer las características y limitaciones de dicho cribado que aparecen recogidas al inicio del presente documento y que se dará traslado de las mismas a los trabajadores mediante la **“Hoja de información a el/la Participante”** (Anexo I) antes de que tomen la decisión de su inclusión y que la misma podrá ser revocada de forma fehaciente en cualquier momento, sin explicación ni consecuencias.

Todos los participantes en el cribado serán seudonimizados por la Empresa/Entidad mediante un código alfanumérico, de tal forma que los datos personales del individuo no puedan ser conocidos por el equipo investigador.

Para el caso en el cual uno de los individuos resulte positivo para SARS-CoV-2, se notificará por el equipo investigador el código positivo al ………………………………………* y será éste el encargado de solicitar la inversión de la seudonimización, de tal forma que pueda notificarle a la persona y a Salúd Pública el resultado positivo, así como a la Empresa/Entidad si estuviese indicado.

*Los resultados sólo pueden ser informados al Servicio, Equipo o Médico responsable de la Salud de la persona en la Empresa / Entidad solicitante.

En…………... a….. de………... de…....

Firmado:

**Anexo I**

HOJA DE INFORMACIÓN AL/A LA PARTICIPANTE ADULTO/A EN EL CRIBADO

PREVENTIVO MEDIANTE TÉCNICAS DE PROCESAMIENTO AGRUPADO “POOLING

Este documento tiene por objeto ofrecerle información sobre el cribado preventivo de personas asintomáticas mediante técnicas de agrupamiento de muestras “Pooling” que se ha iniciado en su empresa y en que se le invita a participar. Este estudio fue aprobado por el Comité de Ética de la Investigación de Galicia.

Si decide participar en el mismo debe leer antes este documento y puede solicitar toda la información adicional que que precise para comprender los detalles sobre el mismo. Si así lo desea puede llevarse el documento, consultarlo con otras personas, y tomarse el tiempo necesario para decidir si participar o no.

La participación en este estudio es completamente voluntaria. Ud. puede decidir no participar o, si acepta hacerlo, cambiar de opinión en cualquier momento sin obligación de dar explicaciones. Esta decisión no afectará en ningún modo a la relación con su empresa ni conllevará ninguna consecuencia ni menoscabo de sus derechos.

Mediante este cribado preventivo se pretende evaluar la presencia/ausencia del virus SARS-CoV-2 causante de la COVID-19 en las muestras de los trabajadores de su empresa como estrategia de control de la transmisión del virus. Si decide participar, usted sólo debe ponerlo en conocimiento de su empresa y esta le dirá cuándo y cómo se realizará la toma de muestra.

Su participación no implica molestias adicionales de las derivadas de la propia toma de muestra.

Los beneficios directos de su participación en el cribado son inciertos, pues dependen de la aparición de algún caso. Este cribado pretende encontrar a personas sanas con posibilidad de contagiar de forma rápida para poder adoptar medidas tempranas de control. Así se se podría frenar la extensión del posible brote asociado al resto de la empresa y la población.

En caso de que el resultado de su muestra sea POSITIVO, el Servicio Médico de su empresa se pondrá en contacto con usted y se le indicaran los pasos que debe seguir.

En caso de resultado negativo, el resultado será registrado en la historia clínica del Servicio Médico de su empresa de la manera habitual, pudiendo usted reclamarlo. La empresa puede instaurar cualquier otra medida adicional de comunicación de resultados con sus trabajadores.

Es muy importante que tenga en cuenta que un resultado negativo no excluye completamente la infección por el Coronavirus, por lo que debe mantener de forma estricta las medidas preventivas adoptadas en su empresa y decretadas por las autoridades sanitarias.

Esta prueba no es diagnóstica, por lo que en caso de tener sospecha de infección por Coronavirus debe acudir a su Centro de referencia para realizar los estudios oportunos.

Muchas gracias por su atención.

**FOLLA DE INFORMACIÓN A O/A PARTICIPANTE ADULTO/A NO CRIBADO PREVENTIVO MEDIANTE TÉCNICAS DE PROCESAMENTO AGRUPADO “POOLING”**

Este documento ten por obxecto ofrecerlle información sobre o cribado preventivo de persoas asintomáticas mediante técnicas de agrupamento de mostras “Pooling” que se iniciou na súa empresa e en que se lle convida a participar. Este estudo foi aprobado polo Comité de Ética da Investigación de Galicia.

Se decide participar no mesmo debe ler antes este documento e pode solicitar toda a información adicional que precise para comprender os detalles sobre o mesmo. Se así o desexa pode levar o documento, consultalo con outras persoas, e tomarse o tempo necesario para decidir se participar ou non. A participación neste estudo é completamente voluntaria. Vde. pode decidir non participar ou, se acepta facelo, cambiar de opinión en calquera momento sen obrigación de dar explicacións. Esta decisión non afectará en ningún modo á relación coa súa empresa nin conlevará ningunha consecuencia nin menoscabo dos seus dereitos.

Mediante este cribado preventivo preténdese avaliar preséncia/ausencia do virus SARS-CoV-2 causante da COVID-19 nas mostras dos traballadores da súa empresa como estratexia de control da transmisión do virus. Se decide participar, vostede só debe poñelo en coñecemento da súa empresa e esta diralle cando e como se realizará a toma de mostra.

A súa participación non implica molestias adicionais das derivadas da propia toma de mostra.

Os beneficios directos da súa participación no cribado son incertos, pois dependen da aparición dalgún caso. Este cribado pretende atopar a persoas sas con posibilidade de contaxiar de forma rápida, para poder adoptar medidas temperás de control. Así se se podería frear a extensión do posible brote asociado ao resto da empresa e da poboación.

No caso de que o resultado da súa mostra sexa POSITIVO, o Servizo Médico da súa empresa poñerase en contacto con vostede e indicásenselle os pasos que debe seguir.

En caso de resultado negativo, o resultado será rexistrado na historia clínica do Servizo Médico da súa empresa da maneira habitual, podendo vostede reclamalo. A empresa pode instaurar calquera outra medida adicional de comunicación de resultados cos seus traballadores.

É moi importante que teña en conta que un resultado negativo non exclúe completamente a infección polo Coronavirus, polo que debe manter de forma estrita as medidas preventivas adoptadas na súa empresa e decretadas polas autoridades sanitarias.

Esta proba non é diagnóstica, polo que en caso de ter sospeita de infección por Coronavirus debe acudir ao seu Centro de referencia para realizar os estudos oportunos.

Moitas grazas pola súa atención.
