# Supplementary figures and images for: Assessment of the Effective Sensitivity of SARS-CoV-2 Sample Pooling Based on a Large-Scale Screening Experience: Retrospective Analysis
Source: JMIR Public Health Surveill. 2024 Sep 24;10:e54503. doi: 10.2196/54503 (PMC11462102; doi:10.2196/54503)

**Supplementary Material**

**Fig. S1:** Comparison between and Global vs


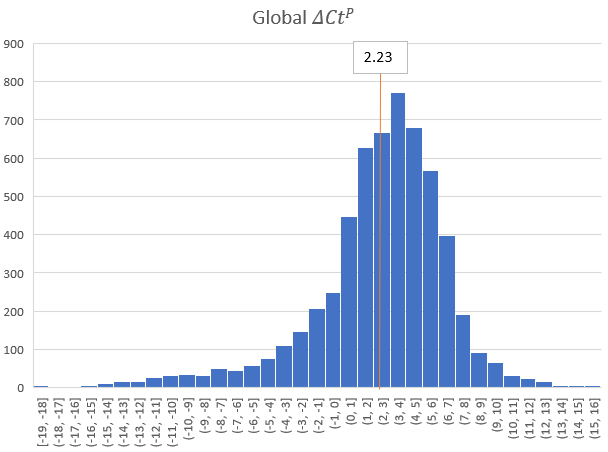


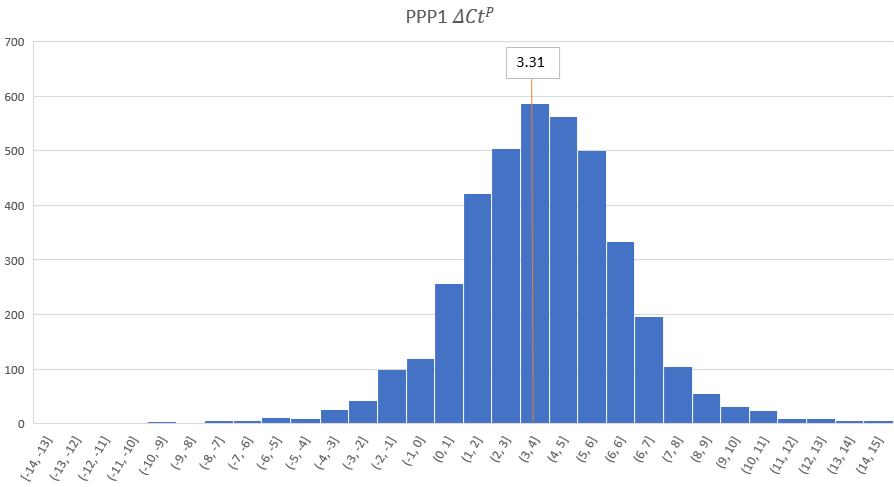


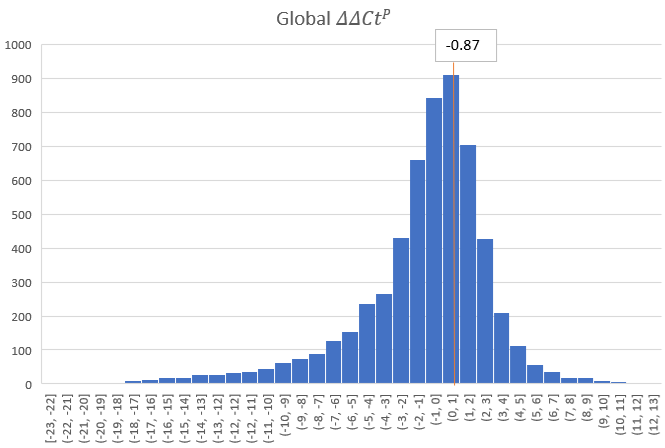


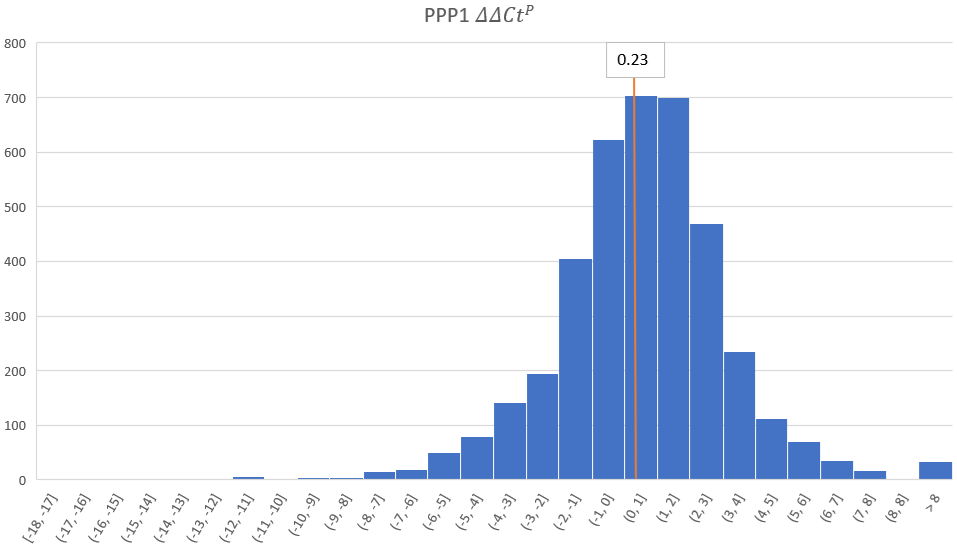

Supplement: Multimedia Appendix 3 [file publichealth_v10i1e54503_app3.docx]
